# miR-34c-3p targets CDK1 a synthetic lethality partner of KRAS in non-small cell lung cancer

**DOI:** 10.1038/s41417-020-00224-1

**Published:** 2020-09-18

**Authors:** Francesco Palma, Alessandra Affinito, Silvia Nuzzo, Giuseppina Roscigno, Iolanda Scognamiglio, Francesco Ingenito, Lola Martinez, Monica Franzese, Mario Zanfardino, Andrea Soricelli, Alfonso Fiorelli, Gerolama Condorelli, Cristina Quintavalle

**Affiliations:** 1 Department of Molecular Medicine and Medical Biotechnology, “Federico II” University of Naples, Naples, Italy; 2 Percuros BV, Zernikedreef 8, 2333 CL Leiden, The Netherlands; 3 IRCCS SDN, Naples, Italy; 4 Flow Cytometry Core Unit, Biotechnology Programme, Spanish National Cancer Research Centre (CNIO), E-28029 Madrid, Spain; 5 Thoracic Surgery Unit, Università degli Studi della Campania “Luigi Vanvitelli,”, Naples, Italy; 6 Institute of Experimental Endocrinology and Oncology (IEOS) G. Salvatore, CNR, Naples, Italy

**Keywords:** Non-small-cell lung cancer, RNAi, Gene expression analysis

## Abstract

Lung cancer is still the leading cause of death by cancer worldwide despite advances both in its detection and therapy. Multiple oncogenic driver alterations have been discovered, opening the prospective for new potential therapeutic targets. Among them, KRAS mutations represent the most frequent oncogene aberrations in non-small cell lung cancer (NSCLC) patients with a negative prognostic impact, but effective therapies targeting KRAS are not well characterized yet. Here, we demonstrate that the microRNA miR-34c-3p is a positive prognostic factor in KRAS-mutated NSCLC patients. Firstly, looking at the TGCA dataset, we found that high miR-34c-3p expression correlated with longer survival of KRAS-mutated NSCLC patients. In vitro assays on immortalized and patient-derived primary NSCLC cells revealed that miR-34c-3p overexpression increased apoptosis and lowered proliferation rate in KRAS^mut^ cells. Computational analysis and in vitro assays identified CDK1, one of the most promising lethal targets for KRAS-mutant cancer, as a target of miR-34c-3p. Moreover, the combination of CDK1 inhibition (mediated by RO3306) and miR-34c-3p overexpression resulted in an additive effect on the viability of KRAS^mut^-expressing cells. Altogether, our findings demonstrate that miR-34c-3p is a novel biomarker that may allow tailored treatment for KRAS-mutated NSCLC patients.

## Introduction

Non-small cell lung cancer (NSCLC) represents one of the two major histological types of lung cancer [1, 2]. Over the past years, it has become evident that NSCLC comprises common genetically driven alterations in oncogenes, such as EGFR (Epidermal Growth Factor Receptor, frequency 10–35%) and KRAS (frequency 15–25%) [3, 4]. Although traditional therapeutic strategies have been improved and target therapies, such as tyrosine kinase inhibitors (TKIs) and EGFR inhibitors, have been successfully used in clinical practice, the overall five-year survival rate for lung cancer remains lower than 16% [5]. Early diagnosis and detection of the lesions and tumour heterogeneity remain huge problems to be addressed. Somatic KRAS mutations are prevalent in many cancers, such as colorectal and pancreatic cancer [6, 7], and leukaemia [8]. In NSCLC, activating mutations are generally limited to adenocarcinoma and are almost always mutually exclusive of EGFR and BRAF mutations. In contrast to EGFR mutations, KRAS mutations are more frequent in males and smokers and are also less commonly identified in some NSCLC subtypes, such as squamous carcinomas and large cell carcinoma [9, 10]. Mutations are often G>T transversions, and hotspots have been found in the GTPase domain of the gene in codons 12, 13 and 61. Mutations in this area can lead to interference with the GTPase activity and reduced negative regulation. The mutational status of KRAS appears, in general, to be associated with poor survival, prognosis, and response to most systemic therapies [11, 12]. KRAS-mutated tumours have intrinsic resistance to EGFR TKIs, such as gefitinib or erlotinib [13]. Despite KRAS mutation being one of the best candidates for the development of tailored anticancer molecules, until now all attempts have been unsuccessful [5]. One of the most promising strategies to block KRAS-mutant oncogenesis is the concept of synthetic lethality, in which suppressed or inhibited genes or proteins cause cell death only in the presence of a cancer-specific alteration. Several synthetic targets for KRAS have been uncovered through genetic screening, such as CDK1, TPX2, SCD1, RAC1 and GATA2 [14].

miR-34c is one of the three members of the miR-34 family: it shares a primary sequence with miR-34b, whereas miR-34a has a specific sequence. This family has been reported deregulated in different carcinomas, such as breast, colorectal, and lung cancers [15, 16]. In particular, low expression of the miR-34 family correlates with poor prognosis in NSCLCs, and miR-34a has just entered a phase I clinical trial in NSCLC patients with mutated KRAS [17]. However, less is known about the association of miR-34c expression and KRAS mutation.

Here, we show that miR-34c-3p expression correlates with better overall survival in NSCLC patients bearing KRAS mutations *vs*. no mutation. Moreover, in KRAS-mutated lung cancer cells, miR-34c-3p reduces cell proliferation and increases apoptosis to a greater extent compared to cells expressing wild-type RAS. In addition, for the first time, we show that miR-34c-3p targets CDK1, a synthetic lethality partner of the KRAS oncogene, and enhances the biological effects of RO3306, a CDK1 inhibitor. Finally, an aptamer–miR-34c-3p chimaera is able to specifically deliver miR-34c-3p to NSCLC cells and target CDK1.

## Materials and methods

### TCGA miRNA dataset and patient information

RNA-Seq and miRNA-Seq data of lung cancerous and normal tissues were downloaded from the TCGA database for LUSC and LUAD patients, from the GDC Data Portal (https://gdc-portal.nci.nih.gov/), using TCGA Biolinks package (version 2.12.0) [18] and pre-processed with ad hoc R scripts. The miRNA expression data included a total of 991 ‘tumour tissue’ patients, consisting of 513 LUAD and 478 LUSC samples and a total of 91 ‘normal tissue’ patients, consisting of 46 LUAD and 45 LUSC samples. The miRNAseq data from primary solid tumour with the corresponding normal tissue were considered in a paired design, for a total of 91 patients. The expression levels of 1881 microRNAs were then pre-processed in three steps: (1) removal of microRNAs whose expression levels were zero across all samples for both conditions; (2) normalization of the remaining microRNAs through the upper-quartile method; and (3) log-transformation of the expression data. Kruskal–Wallis test (*p*-value < 0.001) was applied to estimate statistical significance between normal and tumour groups. Incidence of KRAS mutation in lung adenocarcinoma was frequent as already reported [9] (Supplementary Table 1).

### Survival analysis of miR-34c^high^ and miR-34c^low^ groups

To perform survival analysis, we considered 167 patients with KRAS mutations and 79 KRAS wild type patients from 991 TCGA tumour patients. For these groups, we downloaded the clinical characteristics (survival and outcome) from the TCGA data portal and selected only 142 KRAS-mutated patients and 29 KRAS-wt patients with available survival data. We calculated hsa-miR-34c distribution from normalized read count for all KRAS-mutated patients and then we divided them into two groups: ‘low’ and ‘high’. We defined ‘low’ as the set of patients with microRNA expression lower or equal to the 25th percentile of distribution, and ‘high’ as those with microRNA expression greater or equal to the 75th percentile of distribution. The difference in survival outcome between “low” and “high” groups was estimated by log rank Mantel-Cox test and plotted by Kaplan–Meier curve. A *P*-value of ≤0.05 was considered statistically significant and outliers were filtered out. The analyses were performed using Survival (version 2.43) and Survminer (version 0.4.4) R packages.

### Correlation analysis of hsa-miR-34c and CDK1 expression

We obtained CDK1 gene expression quantification (FPKM-Upper Quartile) for LUSC and LUAD patients from TCGA data. We considered 149 from 167 KRAS-mutated patients with available CDK1 mRNA expression data. We performed a Shapiro-Wilk test on these patients to verify not-normal data distribution and then did correlation analysis. A Spearman’s rho statistic was used to estimate a rank-based measure of association between has-miR-34c expression and CDK1 gene expression. Outliers were filtered out from the analysis. The correlation analysis was performed using psych (version 1.8.12) R package.

### Cell culture and reagents

All the human NSCLC cell lines were purchased from American Type Culture Collection (ATCC). A549, CALU-1, and HCC827 cells were grown in RPMI-1640 medium supplemented with 10% heat-inactivated fetal bovine serum (FBS), 2 mM l-glutamine, and 100 U/ml penicillin/streptomycin [19]. For primary cell-culture experiments, human lung biopsies were obtained from Azienda Ospedaliera Universitaria, Università degli Studi della Campania, Naples, Italy, and processed as indicated elsewhere [20]. For transient transfection of miRNAs, cells were seeded in 6-well plates one day ahead, grown to 50–70% confluence, and then transfected with 100 nM (final concentration) of pre-miR-34c-3p, pre-miR-negative control #1 (miR-NC), anti-miR-34c-3p, or anti-miR-NC (Ambion®, ThermoFisher Scientific, Milan, Italy), and 1 µg of KRAS^G12V^ (KRAS^mut^) plasmid (a kind gift of Prof. Gabriella De Vita, University of Naples Federico II) using Lipofectamine 3000 (ThermoFisher Scientific, Milan, Italy), according to the manufacturer’s protocol. RO3306 was purchased by Sigma Aldrich (Milan, Italy) and used at final concentration of 9 μM.

### RNA extraction and real-time PCR

Total RNAs (microRNA and mRNA) were extracted using Trizol reagent (Invitrogen, Life Technologies, Monza, Italy), according to protocols recommended by the manufacturer. In all, 500 ng of total RNA was retrotranscribed using SuperScript III Reverse Transcriptase (ThermoFischer Scientific, Milan, Italy). For quantization, the 2(−∆∆CT) method was used. Experiments were carried out in triplicate for each data point, and data analysis was performed by using Applied Biosystems StepOne Plus™ Real-Time PCR Systems (ThermoFisher Scientific). The following primers were used: CDK1 fw: 5′-GGGGTCAGCTCGTTACTCAA-3′, CDK1 rv: 5′-TGACATGGGATGCTAGGCTT-3′; B-ACTIN fw: 5′-TGCGTGACATTAAGGAGAAG-3′, B-ACTIN rv 5′- GCTCGTAGCTCTTCTCCA- 3′.

### DNA extraction and mutation testing by real-time PCR

NSCLC patient-derived cell lines were subjected to DNA extraction using QIAamp DNA Mini Kit (Qiagen, Milan, Italy). The extraction of genomic DNA was performed according to the manufacturer’s instructions. Quality and quantity of the extracted DNA samples were measured using NanoDrop (Life Technologies, Carlsbad, CA, USA). Therascreen KRAS RGQ PCR kit (IVD) was performed using 50 ng of DNA input. The DNA was amplified using Amplification Refractory Mutation System (ARMS) technology and detected using Scorpions dual-primer probes. We analysed seven KRAS mutations in codon 12 (c.34G>A, p.Gly12Ser; c.35G>A, p.Gly12Asp; c.34G>C, p.Gly12Arg; c.34G>T, p.Gly12Cys; and c.35G>T, p.Gly12Val) and codon 13 (c.38G>A, p.Gly13Asp) of KRAS’s exon 2,according to the assay design.

### Protein isolation and western blotting

Cells were washed twice in ice-cold PBS, and lysed in lysis buffer (containing 50 mM HEPES pH 7.5, 150 mM NaCl, 1% glycerol, 1% Triton X100, 1.5 mM MgCl2, 5 mM EGTA, 1 mM Na_3_VO_4_, and 1x protease inhibitor cocktail). Protein concentration was determined by the Bradford assay (BioRad, Milan, Italy) using bovine serum albumin (BSA) as the standard. Proteins were resolved on polyacrylamide gel (10–12%) as reported elsewhere [20, 21]. Primary antibodies used were: anti-KRAS (1/1000; (F234) sc-30 Santa Cruz Biotechnology, MA, USA), anti-CDK1(1/1000; 77055 Cell Signaling Technology), anti-β Actin (1/10000; A5441Sigma Aldrich, Milan, Italy), and Vinculin (1/1000 13901; Cell Signaling Technology).

### In vitro proliferation and apoptosis assays

Cell viability was evaluated with the CellTiter 96 AQueous One Solution Cell Proliferation Assay (Promega, Milan, Italy), according to the manufacturer’s protocol. After 30 min. of incubation, the plates were analysed on a Multilabel Counter (Bio-Rad, Milan, Italy). The Annexin V method was used to examine HCC827 and A549 cell apoptosis. Briefly, cells were plated at 2.5 × 10^5^ cells/well in six-well plates, incubated for 24 h, and then transfected. Cells were harvested, washed with PBS, stained with 5 μl of 20 μg/ml propidium iodide (PI) containing 1 mg/ml RNase in PBS for 20 min, and conjugated with Annexin V-FITC using a Annexin V-FITC kit (BD Biosciences, MD, USA) according to the manufacturer’s protocol. Apoptotic cells were defined as Annexin V-positive/PI-positive and Annexin V-positive/PI-negative cell populations among total gated cells. Cells were analysed using a BD AccuriTM C6 flow cytometer (BD Biosciences) and analysed using the BD AccuriTM C6 software. Data are presented as mean values ± SD of at least two independent experiments. Statistical significance was accepted for *p* values < 0.05 by ANOVA test versus the control.

### Analysis of caspase3/7 activity

Caspase-3/7 activity was measured by using the Caspase Glo-3/7 assay system (Promega, Milan, Italy). Briefly, cells (5 × 10^5^ cells/10 ml) were seeded in 96-well plates and after 24 h transfected with microRNA. Subsequently, cells were incubated with 100 μl of Caspase GLO substrate. Finally, the luminescence of each sample was measured by using Luciferase assay system (Promega).

### Luciferase reporter assay

Putative binding sites for miR-34c-3p on the 3′ UTR of CDK1 were predicted by Targetscan Version 7.2 software (http://www.targetscan.org). The identified region was cloned in pmiRglo plasmid (Promega) using the following primer: FW: 5′-CGCAAATTGTGGATTGCAACCCTTTAGTGATTTACGACCAGT-3′; RV: 5′-CTAGACTGGTCGTAAATCACTAAAGGGTTGCAATCCACAATTTGCGAGCT-3′.A549 and HCC827 cells (5000 cells per well) were seeded in a 96-well plate and co-transfected with 1 µg of 3′UTR CDK1 plasmid (and miR-34c-3p or miR-NC), using Lipofectamine 3000. Both firefly and Renilla luciferase expression was measured 24 h and 48 h post-transfection using the Dual Luciferase Assay (Promega), according to the manufacturer’s instructions.

### Immunofluorescence microscopy

Cells were seeded onto glass coverslips at 5 × 10^4^ cells/ml in six-well plates overnight, and then fixed with 4% paraformaldehyde in PBS for 30 min at room temperature (RT). Fixed cells were washed with PBS and permeabilized with 1% Triton X-100 in PBS for 10 min. After washing with PBS, the coverslips were incubated with anti-cleaved-caspase-3 antibody (#9669, Cell Signaling) diluted in 5% BSA/PBS for 1 h at RT. They were then washed twice with 0.02% Tween 20 and 1% BSA in PBS, followed by incubation with Alexa Fluor 488-conjugated anti-rabbit (BD Bioscience) for 30 min at RT. After washing 3× with 0.02% Tween 20 and 1% BSA in PBS, the cover slips were mounted with Vectorshield (Vector Labs, CA, USA) containing DAPI. The cover slips were then washed once before incubation with Alexa Fluor 488-conjugated anti-cleaved-caspase-3. After washing 3× with 0.02% Tween 20 and 1% BSA in PBS, the cover slips were mounted. Cells were examined under a Ziess LSM 510 laser scanning fluorescence confocal microscope.

### Aptamer-miR Complexes

Sequences of Gl21.T scrambled and Gl21.T-miR34c-3p have been reported before [20]. For complex generation, (1) 5 μM of miR-34c-3p strands (miR-34c guide and miR-34c passenger sticky) were incubated at 95 °C for 15 min, 55 °C for 10 min, and RT for 20 min, in Binding Buffer 10x (200 mM HEPES [pH 7.4], 1.5 M NaCl, 20 mM CaCl2); (2) stick aptamer was refolded (5 min 85 °C, 3 min on ice,10 min at 37 °C); (3) equal amounts of stick aptamer and passenger-guide duplex were then annealed by incubating together at 37 °C for 30 min. The annealing efficiency was evaluated on a 12% non-denaturing polyacrylamide gel. For all experiments, treatments with SCRA/miR34c or GL21.T/miR34c chimaera were performed at 400 nM (final concentration).

### Statistical analysis

Data are presented as mean ± standard deviation (SD). For comparisons between two groups, Student’s *t* test was used to determine differences between mean values for normal distribution, for multiple comparisons ANOVA was applied. Variances between the analysed groups were similar. Data were analysed for significance using GraphPad Prism 6 software (San Diego, CA, USA). *P*-values less than 0.05 were considered significant.

## Results

### miR-34c is downregulated in NSCLC tissue

**Fig. 1:**
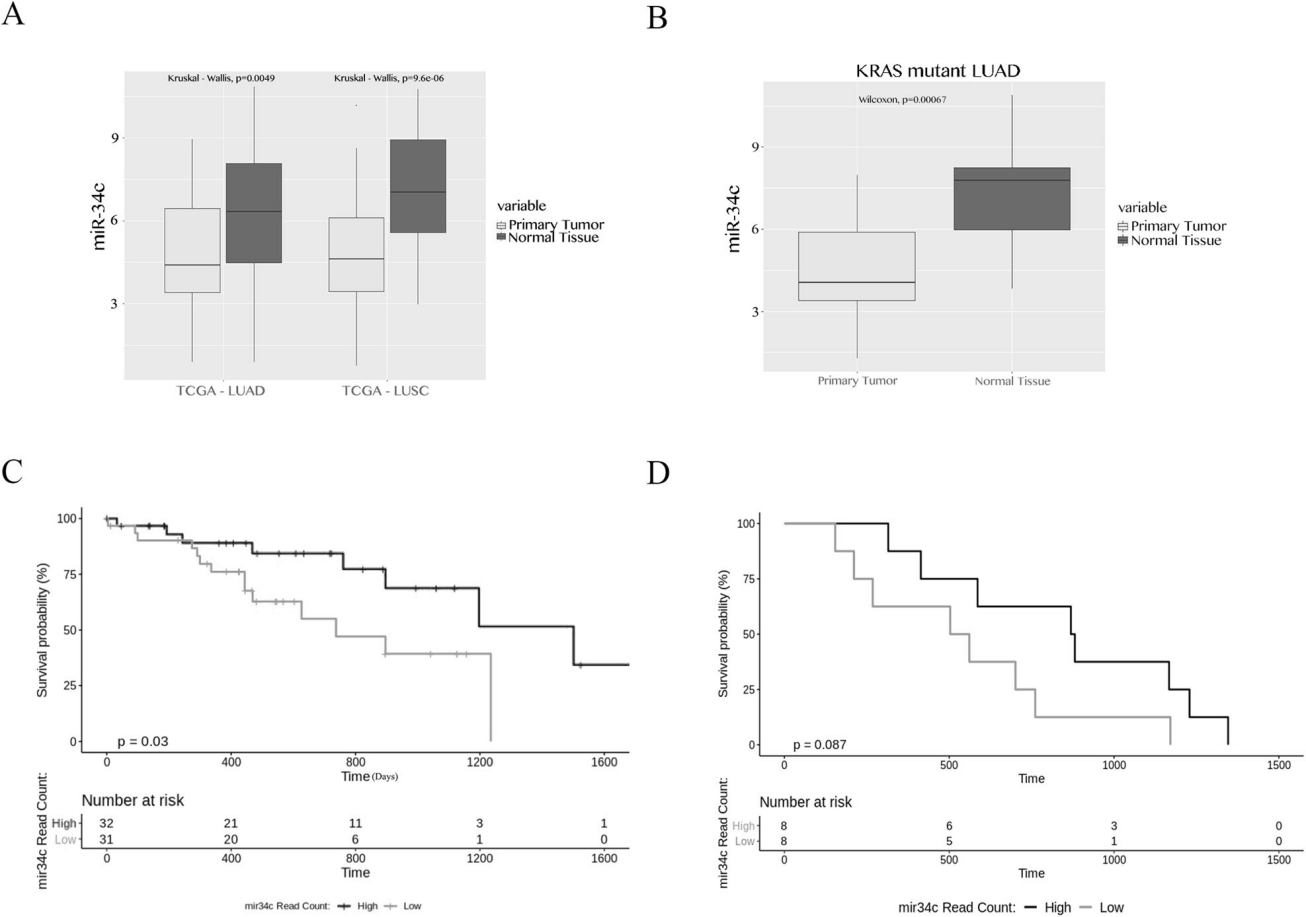
miR-34c expression in lung adenocarcinoma. **a** TCGA database analysis of miRNA-seq data of lung cancer and normal tissues. miR-34c expression in 91 paired (normal vs tumour) lung adenocarcinoma stratified in 46 LUAD and 45 LUSC patients. Kruskal–Wallis test was applied to estimate statistical significance (*p*-value < 0.001). **b** miR-34c expression in 15 paired KRAS-mutated lung adenocarcinoma (LUAD patients) and normal tissues by bioinformatics analysis of the TCGA database. Wilcoxon test was applied to estimate statistical significance (*p*-value ≤ 0.05). **c** Survival analysis at five years of 33 lung adenocarcinoma patients with KRAS mutation stratified into low and high expression groups for miR-34c: miR-34c low was assigned for miRNA levels lower or equal to the 25th percentile of the distribution; miR-34c high for values greater or equal to the 75th percentile of distribution. The difference in the survival outcome between ‘low’ and ‘high’ groups was estimated by log rank Mantel-Cox test and plotted on a Kaplan–Meier curve (*p*-value ≤ 0.05). **d** Survival analysis at five years of 29 (8 High, 8 Low and 13 between 25th percentile and 75th percentile of the distribution that have not been considered) lung adenocarcinoma patients without KRAS mutation stratified into low and high expression groups for miR-34c: miR-34c low was assigned for miRNA levels lower or equal to the 25th percentile of the distribution; miR-34c high for values greater or equal to the 75th percentile of distribution. The difference in the survival outcome between ‘low’ and ‘high’ groups was estimated by log rank Mantel-Cox test and plotted on a Kaplan–Meier curve.

In order to characterize the involvement of miR-34c in NSCLC, we performed bioinformatics analysis on a cohort of NSCLC patients (91 tumour samples, 46 with lung adenocarcinoma (LUAD) and 45 with lung squamous cell carcinoma (LUSC) and matched normal samples from the GDC Data Portal (https://gdc-portal.nci.nih.gov/). We interrogated the TGCA data base, and, as reported previously by Russo et al. [20]. This analysis confirmed that miR-34c was significantly downregulated in LUAD/LUSC groups *vs*. matched, non-tumour counterparts (Fig. 1a). We then investigated miR-34c expression in KRAS-mutated NSCLC tissue *vs*. control normal tissue. We observed that in 15 paired LUADs with a KRAS mutation, the expression of miR-34c was downregulated *vs*. normal tissue (Fig. 1b). Moreover, we further interrogated the TGCA data base to assess miR-34c-3p and miR-34c-5p expression levels in LUAD/LUSC groups *vs*. matched, non-tumour counterparts in wild-type KRAS patients. Even if there were only 8 paired patients with data available for miR-34c-3p and miR-34c-5p, we observed a strong reduction of both miRNAs in tumour samples *vs*. matched, non-tumour samples (Supplementary Fig. 1a). The same results were also observed analysing miR-34c-3p and miR-34c-5p expression levels in 14 paired LUAD KRAS^mut^ tumour samples *vs*. matched, non-tumour counterparts (Supplementary Fig. 1b), confirming the protective role of miR-34c.

More interestingly, when stratifying KRAS-mutated patients into two groups on the basis of miR-34c expression (33 patients with miR-34c^high^ and 33 patients with miR-34c^low^), survival analysis indicated that high expression was associated with longer survival at 5 years (Fig. 1c).On the contrary, in KRAS wild type patients we did not observe any difference in 5 years survival (Fig. 1d).

### KRAS mutation status in NSCLC cell lines

We then investigated by different means the mechanisms underlying the higher survival rate of miR-34c-3p^high^ KRAS-mutated patients. First, we checked the mutational status of KRAS in primary and continuous cell lines, using the TheraScreen KRAS RGQ PCR kit. As shown in Table 1, the cohort of primary cell lines available and the continuous cell line HCC827 were negative for KRAS mutation, whereas A549 and Calu-1 cells were KRAS-mutated (KRAS c.34G>A and KRAS c34G>T, respectively), as already reported by ATCC and others [22, 23]. At the same time, miR-34c-3p levels were analysed by RT-qPCR in the different cell lines (Supplementary Fig. 2a).

**Table 1:** KRAS mutational profile in NSCLC cells.

No.	TheraScreen mutation Ct	TheraScreen mutation
PT#2	N/A	WT
PT#13	N/A	WT
PT#18	N/A	WT
A549	30.4	KRAS c.34G>T
HCC827	N/A	WT
Calu-1	29.7	KRAS c.34G>T

TheraScreen results on primary NSCLC (PT#2, PT#13 and PT#18) and immortalized A549, HCC827 and Calu-1 cell lines.

### Overexpression of miR-34c-3p impairs in vitro growth of KRAS-mutated NSCLC

**Fig. 2:**
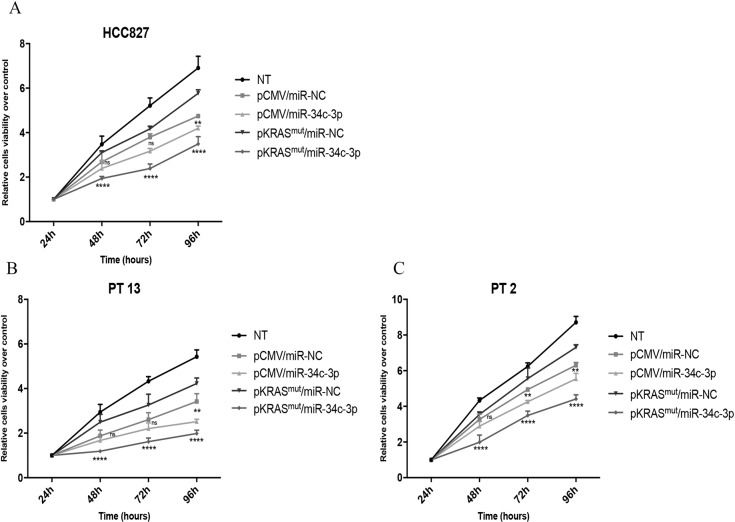
Overexpression of miR-34c-3p impairs proliferation of KRAS-mutated NSCLC cells. **a** HCC827 cells were transfected with miR-34c-3p and a KRAS-mutant vector, and proliferation assessed at 24, 48, 72 and 96 h by MTT assay. Cells transfected with an empty vector and/or a scrambled miRNA were used as control. Two-way ANOVA was used for comparison (pCMV/miR-NC vs pCMV/miR-34c-3p, and KRAS^mut^/miR-NC vs KRAS^mut^/miR-34c-3p). **b**, **c** Primary epithelial NSCLC cells from patients #13 and #2 were co-transfected with miR-34c and KRAS mutant vector. Proliferation was analysed by MTT assay at 24, 48,72, and 96h and compared to cells transfected with an empty vector and a scrambled miRNA. Two-way ANOVA was used for comparison (pCMV/miR-NC vs pCMV/miR-34c-3p, and KRAS^mut^/miR-NC vs KRAS^mut^/miR-34c-3p). Experiments were performed in technical triplicate and repeated three times. Bar graphs indicate mean value ± SD (*p ≤ 0.05; **p ≤ 0.01; ****p ≤ 0.0001).

Our previous data on the KRAS-mutated cell lines A549 and CALU-1 suggested that miR-34c-3p reduces cell proliferation [20]. We observed the same result in wild-type KRAS HCC827 cells (Fig. 2a). To further assess the role of miR-34c-3p and KRAS signalling, primary cell lines (PT#13, PT#2) and HCC827 cells were co-transfected with KRAS^mut^ and miR-34c-3p, and cell viability assessed. As shown in Fig. 2b, c, miR-34c-3p decreased viability also of KRAS^mut^cells. The expression of KRAS was verified by western blotting (Supplementary Fig. 2b). These results confirmed that miR-34c-3p behaves as a tumour suppressor in NSCLC cells and can overcome KRAS-mutant-induced oncogenesis.

### miR-34c-3p activates apoptosis in KRAS-mutated NSCLC

**Fig. 3:**
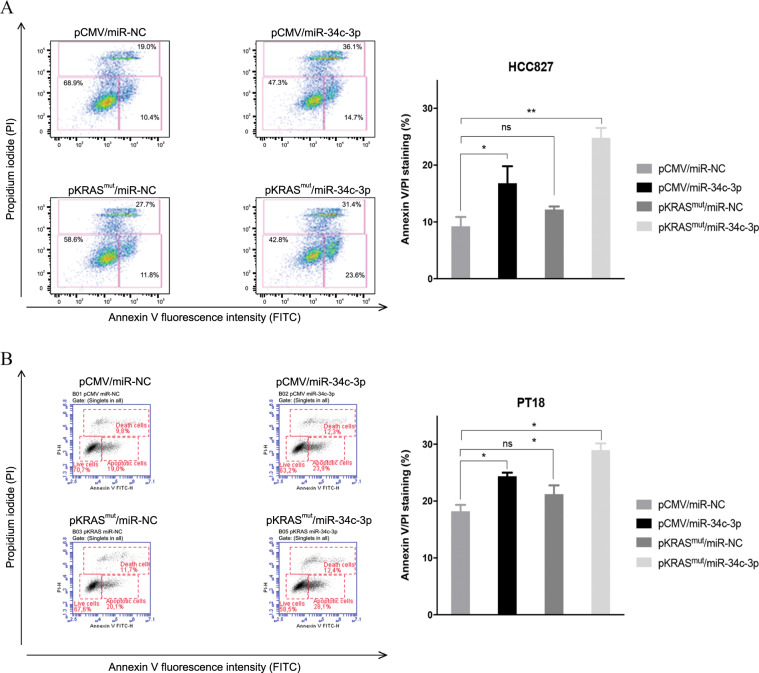
miR-34c-3p overexpression increases apoptosis in KRAS-mutated NSCLC cells. **a-** Annexin-V and propidium iodide staining of HCC827 cells simultaneously transfected with miR-34c-3p and KRAS mutant vector for 48 h. Cells transfected with scrambled miRNA and an empty vector were used as control. The graph on the right reports the quantification (analysed by FlowJo) of the number of live cells and apoptotic cells in each experimental point. Two-way Anova was used for comparison (*p ≤ 0.05; **p ≤ 0.01). **b** Annexin-V and propidium iodide staining of PT#18 cells co-transfected with miR-34c-3p and KRAS mutant vector. Data were analysed with the BD AccuriTM C6 plugin software. ANOVA was used for comparison (*p ≤ 0.05; **p ≤ 0.01). Experiments were performed twice.

Next, we investigated whether miR-34c-3p affects cell death. We analysed apoptosis activation in HCC827 cell lines upon overexpression of miR-34c-3p and KRAS^mut^. Upon 48 h of transfection, cells were solubilized and conjugated with Annexin V-FITC. Flow cytometry analysis (Fig. 3a) revealed that the apoptotic rate was significantly increased in cells transfected with miR-34c-3p vs. controls. Moreover, statistically significant increases in Annexin V+ and PI + apoptotic cells were observed in HCC827 cells expressing miR-34c-3p and KRAS^mut^(Fig. 3a). The same effect was observed for PT#18 primary cell lines co-transfected with KRAS^mut^ and miR-34c-3p (Fig. 3b).

**Fig. 4:**
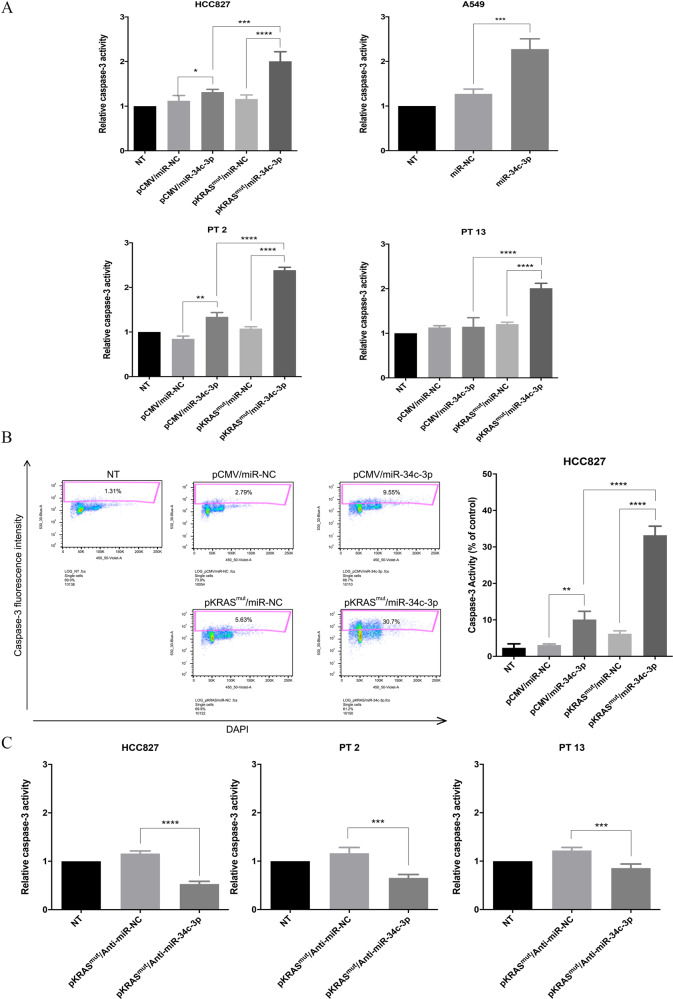
Overexpression of miR-34c-3p induces caspases activation in KRAS-mutated NSCLC cells. **a** Caspase-3 activation measurement in HCC827, A549, and primary epithelial NSCLC cell lines (PT #2 and PT #13) upon 48 h of transfection with miR-34c-3p and KRAS mutant vector. Bar graphs indicate mean value ± SD and the p value was calculated by ANOVA test. Experiments were performed in technical duplicate and repeated three times. **b** FACS analysis of caspase-3 activation of in HCC827 cells upon simultaneous transfection of miR-34c-3p and KRAS mutant vector. Cells transfected with an empty vector and a scrambled miRNA were used as control. Bar graphs indicate mean value ± SD and the *p*-value was calculated by ANOVA test. Experiments were performed twice. **c** Caspase-3 activation measured in HCC827 and primary epithelial NSCLC cell lines (PT #2 and PT #13) upon anti-miR-34c-3p transfection (an anti-scrambled miRNA was used as control). Experiments were performed in technical duplicate and repeated three times. Bar graphs indicate mean value ± SD and the *p-*value was calculated by ANOVA (*p ≤ 0.05; **p ≤ 0.01; ***p ≤ 0.001;****p ≤ 0.0001).

To address the involvement of caspase-3 activation in the different groups, we used a caspase-Glo3/7 assay. In HCC827 and A549 continuous cell lines, and in two patient-derived cell lines (PT#2 and PT#13), caspase-3 activity was higher in the KRAS^mut^- and miR-34c-3p-transfected cells (Fig. 4a). The result was confirmed by analysing caspase-3 activation in HCC827 cells by FACS (Fig. 4b). Conversely, anti-miR-34c transfection reduced caspase 3/7 activity, as assessed by the caspase-Glo3/7 assay (Fig. 4c). Moreover, as assessed by immunofluorescence analysis, a larger number of positive cells for FITC-conjugated anti–cleaved caspase 3 monoclonal antibodies were observed in HCC827 cells transiently co-expressing KRAS^mut^and miR-34c-3p (Supplementary Fig. 2c).

### CDK1 is a direct target of miR-34c-3p

**Fig. 5:**
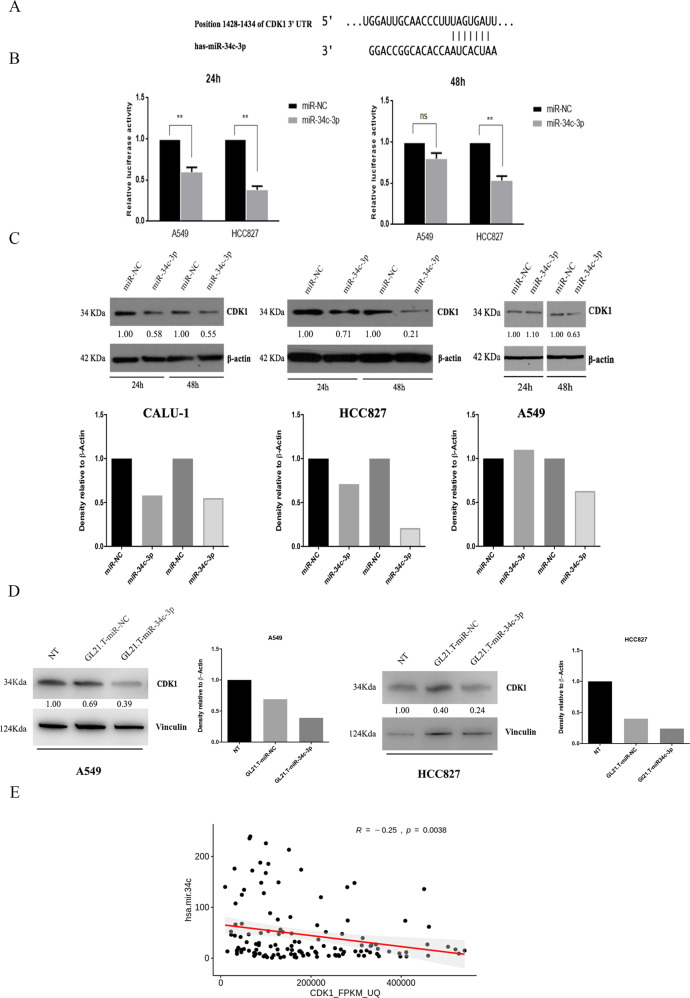
CDK1 is a target of miR-34c-3p. **a** Schematic representation of predicted CDK1mRNA and hsa-miR-34c-3p binding sites in position 1428-1434 of the 3′-UTR. **b** A549 and HCC827 cells were transiently transfected with CDK1-3′ UTR in the presence of miR-34c-3p or a miRNA negative control (miR-NC). Luciferase activity was evaluated 24 and 48 h after transfection. Experiments were performed in technical triplicates and repeated twice. Bar graphs indicate mean value± SD and the p-value was calculated by Student’s *t* test. **c** Western blotting analysis of CDK1 protein after transfection of miR-34c-3p or a negative control in HCC827, A549, and CALU-1 cell lines. Βeta-actin was used as loading control. The experiment was done twice. A representative blot is shown. Bar graphs represent CDK1 densitometry normalized for beta-actin loading. **d** Western blotting for CDK1 protein after treatment with the GL21.T-miR34c-3p chimaera or GL21.T-miR-NC in A549 and HCC827 cells. Vinculin was used as loading control. The experiment was done twice. A representative blot is shown. **e** Correlation analysis of CDK1 mRNA and miR-34c in LUSC and LUAD patients from the TCGA data base. We considered 149 out of 167 KRAS-mutated patients with available CDK1 mRNA expression data. Spearman’s rho statistic was used to estimate a rank-based measure of association between the two entities.

We then investigated possible targets of miR34c-3p with miRNA target prediction algorithms. We identified a putative miR-34c-3p binding site on the 3′ UTR of CDK1 (Fig. 5a), recently reported to be involved in a synthetic lethality pathway of KRAS mutations [14]. To validate whether miR-34c-3p directly binds to the 3′ UTR of CDK1 mRNA, a dual luciferase reporter assay was performed. When HCC827 and A549 cells were transiently co-transfected with CDK1-3’UTR and miRNA-34c-3p, there was a significant and consistent reduction in luciferase activity (>50%) (Fig. 5b). We then assessed the effect of miR-34c-3p transfection on CDK1 protein levels in Calu-1, HCC827, and A549 cells. Exogenous expression of miR-34c-3p induced in these cells a significant reduction of CDK1 (Fig. 5c). Interestingly, the other member of the miR-34 family, miR-34a, did not induce CDK1 downregulation (data not shown).

We then investigated whether targeted delivery of miR-34c-3p into AXL receptor-expressing NSCLC cells induced similar effects on CDK1 expression. The AXL-recognizing aptamer (GL21.T) was conjugated with miR-34c-5p, as previously described [20], and incubated with HCC827 cells. Interestingly, the GL21.T-miR-34c-3p chimaera decreased CDK1 levels (Fig. 5d), supporting the possibility of using aptamer-based methods for targeted delivery of miR-34c-3p to NSCLC cells. Moreover, correlation analysis carried out on 149 KRAS-mutated patients from the GDC Data Portal revealed a significant inverse correlation between CDK1 mRNA and miR-34c-3p (Fig. 5e). These findings suggest that miRNA-34c-3p directly targets CDK1, and that this target may mediate some of miRNA-34c-3p’s effects on NSCLC proliferation and survival.

### miR-34c-3p overexpression acts synergistically with CDK1 inhibition to affect proliferation and apoptosis

**Fig. 6:**
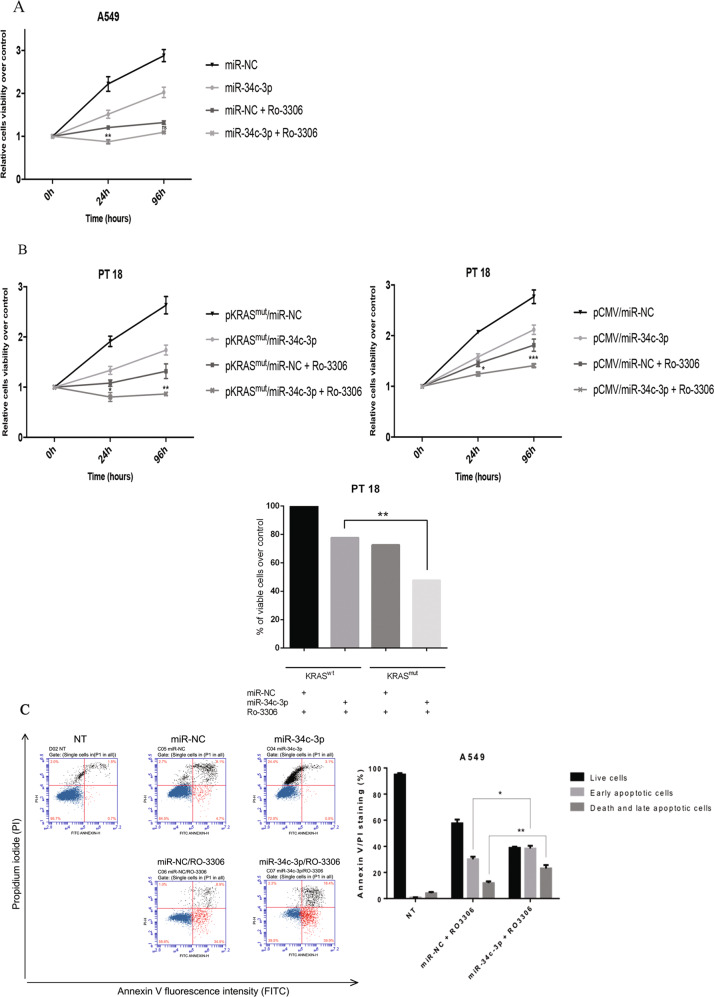
The combination of miR-34c-3p and RO3306 affects proliferation and apoptosis. **a** Proliferation analysis of KRAS-mutant A549 cells transfected with miR-34c-3p and **b** primary epithelial cells from PT#18 co-transfected with miR-34c-3p and a KRAS mutant vector (left panel) or with control vector (pCMV) (right panel), in combination or not with RO3306. Proliferation was analysed after 24 and 96 h of treatment. Experiments were performed in technical triplicates and repeated twice. Data are presented as mean ± SD. Two-way ANOVA was used for comparisons (A549 miR-34c-3p vs miR-34c-3p + R0 = 3306; and PT#18 and KRAS^mut^/miR-NC + RO-3306 vs KRAS^mut^/miR-34c-3p + RO3306). Bar graph represents the percentage of viable cells upon 96 h of transfection, comparing NSCLC cells expressing RAS wild type (pCMV) or KRAS mutated (pKRAS^mut^) **c** Annexin-V and propidium iodide staining of A549 cells transfected with miR-34c-3p and treated with RO3306. Cells transfected with scrambled miRNA were used as control. The graph on the right reports the quantification of the number of live cells and apoptotic cells in each experimental point. Data are presented as mean ± SD. ANOVA was used for statistical analysis. (*p ≤ 0.05; **p ≤ 0.01; ***p ≤ 0.001; ****p ≤ 0.0001). Experiments were performed two times.

There is significant interest in investigating the role of CDK inhibitors in human cancer, including lung cancer. In fact, those drugs may be eventually used as second-line-based therapy in KRAS-mutated patients. We hypothesized that the therapeutic response in those patients may be improved when CDK1 is affected at both protein and activity levels. Therefore, we studied proliferation and apoptosis on cells overexpressing miR-34c-3p and exposed to the CDK1 inhibitor RO3306. To this end, the KRAS-mutated A549 cell line was transfected with miR-34c-3p (or a miRNA control) and the effects of RO3306 on cell proliferation analysed by MTT assay. Experiments on A549 cells transfected with miR-34c-3p indicated that the number of viable cells was clearly reduced *vs*. the negative control; moreover, this effect was potentiated in the presence of RO3306 (Fig. 6a). Apoptosis has been assessed by Annexin-V and propidium iodine staining and FACS analysis (Fig. 6c). Similar results on cell viability were observed in a primary NSCLC cell line (PT#18) co-transfected with KRAS^mut^ and miR-34c-3p (Fig. 6b, left panel). The effect of CDK1 inhibition on cell viability was lower in primary NSCLC cells expressing RAS wild type (Fig. 6b, right panel and histogram).

## Discussion

The miR-34 family, composed of miR-34a, miR-34b, and miR-34c, is a powerful tumour suppressor in several malignancies. It is involved in the regulation of apoptosis, cell cycle, and senescence [17]. It has been reported that miR-34 family members are downregulated in NSCLC and are able to reduce NSCLC cell survival in vitro and in vivo [24]. Recently, miR-34b and miR-34c have been reported to have more powerful anti-tumoural roles in lung adenocarcinoma than does miR-34a [25]. We have also previously reported that miR-34c-3p is downregulated in NSCLC and suppresses tumour growth [20]. Interestingly, Huang W et al. recently showed that miR-34c-3p levels were lower also in exosomes from the serum of NSCLC patients *vs*. healthy controls, showing a significant negative association with disease-free survival [26]. The same finding was also reported for the circulating miR-34 family in NSCLC plasma samples [25, 27].

In lung cancer, KRAS is one of the most frequent genetic alterations, and its expression is associated with pathological features, prognosis, and response to therapy [28, 29].In this manuscript, we show that high expression of miR-34c is correlated with survival in lung-adenocarcinoma patients carrying KRAS mutations. In fact, TGCA data analysis highlighted that high miR-34c levels correlate with a better survival rate (at 5 years) in patients with an underlying KRAS mutation. The study of the role of miR-34c-3p in KRAS NSCLC patients could help identify new prognostic factors and determine novel targets that may allow a tailored treatment for these patients.

Since the discovery of the involvement of KRAS in lung cancer in 1984 by Barbacid’s group [30], much effort has been directed at the better characterization of KRAS in the development of lung cancer [31]. KRAS alterations have been found to be strictly associated with lung adenocarcinoma development and smoking [29]. Oncogenic KRAS can activate up to 10 different pathways, the best characterized of which are the MAPK cascade and the PIK3-AKT-mTOR pathway [32]. All these effectors have been extensively investigated to find new therapeutic strategies for KRAS-mutated adenocarcinoma. Unfortunately, all the attempts made until now have been unsuccessful, driving the necessity to explore alternative strategies for targeting KRAS-driven cancer. Recently, two small molecules named AMG510 and MRTX849 have shown clinical promise for the treatment of NSCLC with KRAS^G12C^ [33]. Therefore, new therapeutic strategies for KRAS-mutated NSCLC are needed.

Despite miR-34 expression being associated with survival in KRAS-mutated adenocarcinoma patients, little is known on the molecular mechanisms by which miR-34c-3p and KRAS interact. We addressed the possibility that synthetic lethality may be a mechanism of mutual interaction between KRAS and miR-34c-3p. Synthetic lethality represents a valid approach for the direct targeting of small and dynamic oncogenic proteins, such as KRAS [34]. Here, for the first time, we describe that miR-34c-3p targets CDK1, a recently reported synthetic lethal target of mutated KRAS [14, 35]. Our findings indicate that miR-34c-3p, but not miR-34a (data not shown), is able to reduce the expression of CDK1 protein.

CDK1 is a cyclin-dependent kinase necessary for controlling cell cycle regulation, and its aberrant expression is responsible for uncontrolled proliferation in cancer cells as well as for genomic and chromosomal instability [36, 37]. Moreover, CDK1 may interfere with the synthetic lethality of other oncogenes, such as MYC in triple-negative breast cancer [38], PARP [39] and PI3-kinase [40]. Interestingly, it has been recently proposed that CDK1 is responsible for apoptosis resistance in BRAF^V600E^ colorectal cancer; indeed, the combination of a CDK1 inhibitor with a MEK/ERK inhibitor enhanced apoptosis [41]. Here, we propose that by targeting CDK1, miR-34c-3p is involved in synthetic lethality in KRAS mutant patients. Indeed, we observed that miR-34c-3p targets CDK1 and has an oncosuppressor effect in mutated KRAS NSCLC cells. However, in NSCLC cells overexpressing mutated KRAS, we observed an additive effect on cell proliferation and apoptosis upon the combination of miR-34c-3p overexpression and exposure to RO03306, a small molecule inhibitor of CDK1.

Moreover, when stratifying KRAS-mutant patients into two groups based on miR-34c expression, survival analysis indicated that high miR-34c expression was associated with longer survival at 5 years. MRX34, a liposomal miR-34a mimic, has been used to assess the use of miR-34 as a therapeutic agent in patients with advanced solid tumours [42]. Even if the approach is extremely interesting, the trial was interrupted because of adverse effects due to unspecific delivery of the drug. Recently, we proposed a new possible tool for targeted delivery in NSCLC [43]: it was based on a chimeric molecule [44] composed of a GL21 aptamer – which recognizes the AXL protein on the surface of NSCLC cells– and miR-34c-3p [20]. Upon internalization, the chimaera increased the levels of miR-34c-3p in AXL-positive cells and reduced survival of NSCLC cells in vitro. In the present work, we demonstrate that the chimaera reduces expression of CDK1 protein, opening a new possible therapeutic scenario for KRAS-mutated adenocarcinoma.

**Fig. 7:**
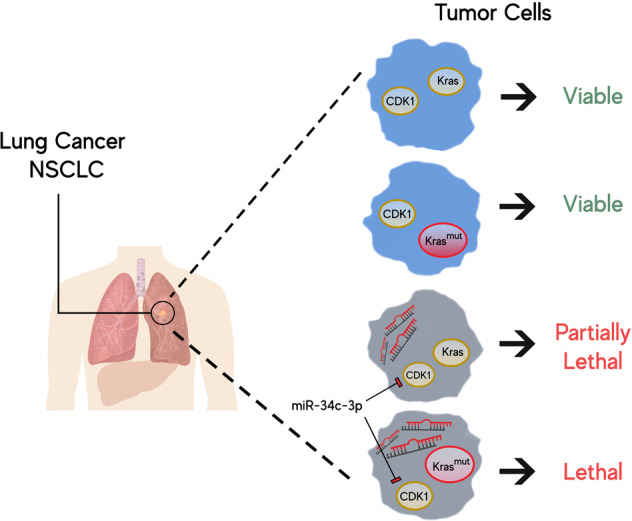
Synthetic lethality as a therapeutic paradigm in NSCLC cancer. The simultaneous mutation of RAS and upregulation of CDK1 confer complete lethality, whereas any other combination of mutations is viable.

Altogether, our findings highlight that, in addition to its already known role as an oncosuppressor miRNA in NSCLC, miR-34c-3p acts in KRAS-mutated NSCLC as a regulator of the CDK1 gene, which is involved in a synthetic lethality pathway (Fig. 7). Thus, miRNA-based treatments could be an option to be translated from the bench to the patient bedside.

## Supplementary information

supplementary information
