# Supplementary material for: miR-34c-3p targets CDK1 a synthetic lethality partner of KRAS in non-small cell lung cancer
Source: Cancer Gene Ther. 2020 Sep 18;28(5):413–26. doi: 10.1038/s41417-020-00224-1 (PMC8119240; doi:10.1038/s41417-020-00224-1)
Supplement: Supplementary file 1 — supplementary information [file 41417_2020_224_MOESM1_ESM.pdf]

Supplementary information files

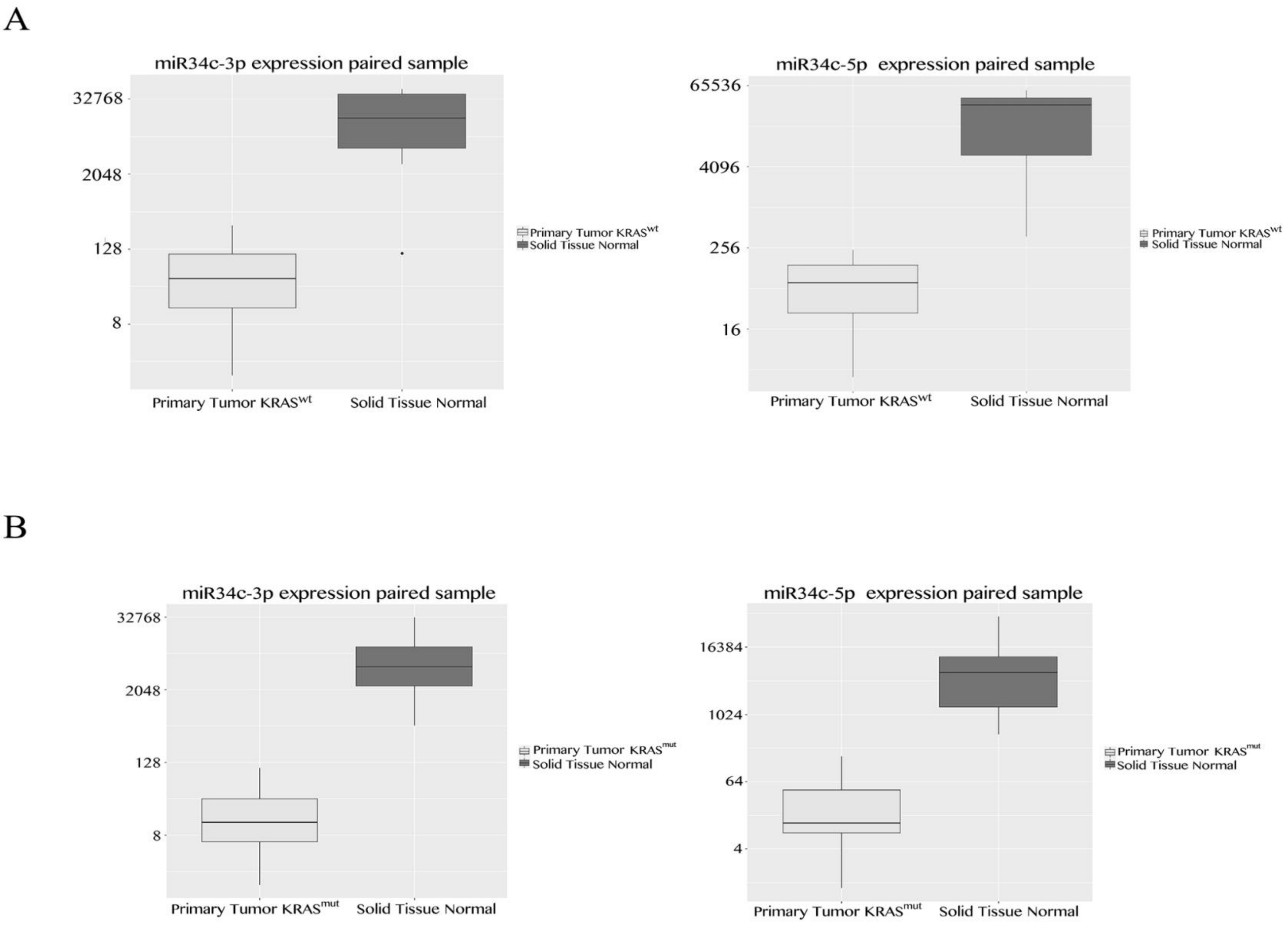

Sup. Fig 1

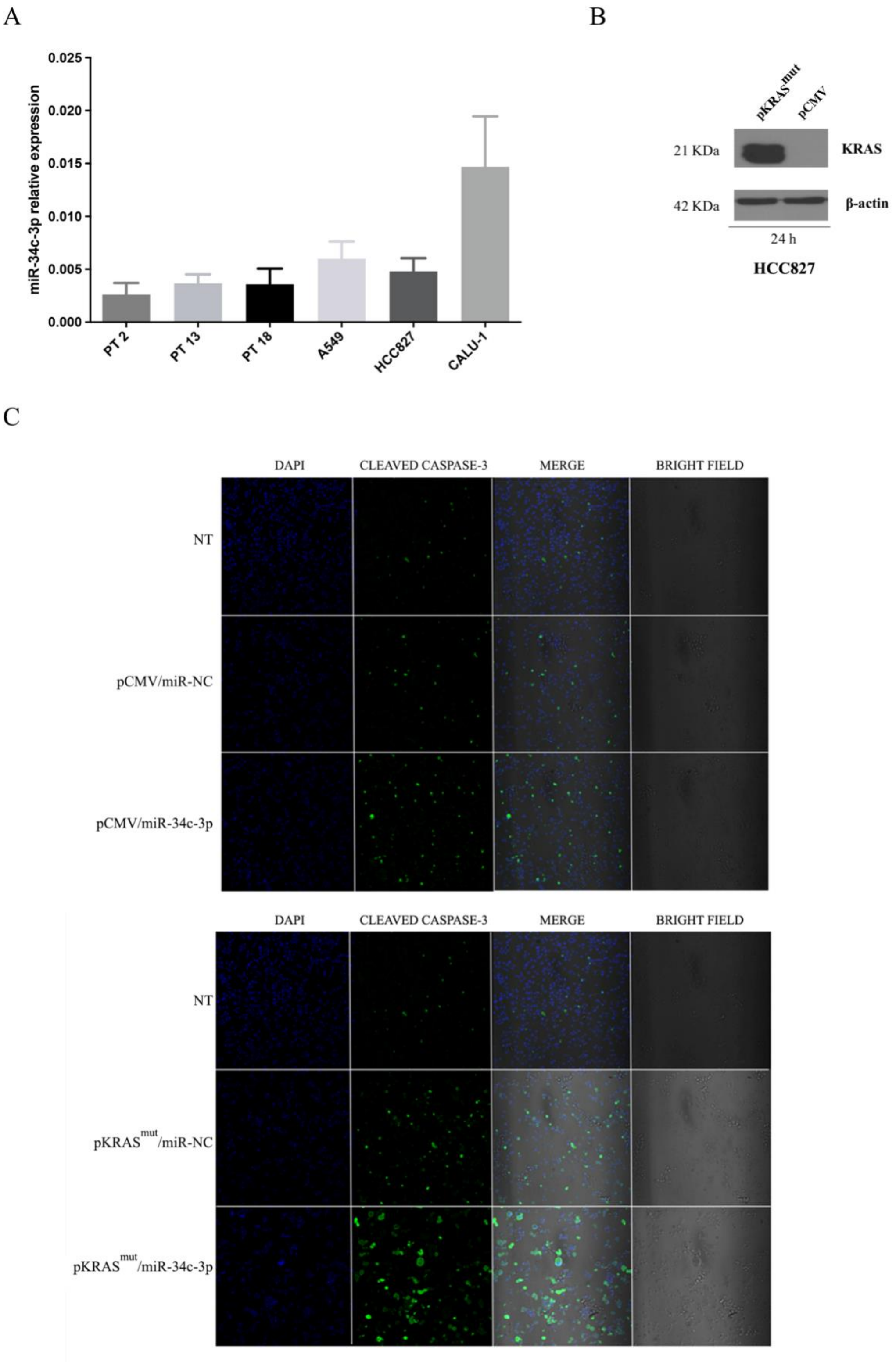
A
miR-34c-3p relative expression
0.025
0.020
0.015
0.010
0.005
0.000
PT 2
PT 13
PT 18
A549
HCC827
CALU-1
B
pKRAS^mut
pCMV
21 KDa
KRAS
42 KDa
β-actin
24 h
HCC827
C
DAPI
CLEAVED CASPASE-3
MERGE
BRIGHT FIELD
NT
pCMV/miR-NC
pCMV/miR-34c-3p
DAPI
CLEAVED CASPASE-3
MERGE
BRIGHT FIELD
NT
pKRAS^mut/miR-NC
pKRAS^mut/miR-34c-3p

Sup. Fig 2

Supplementary Figure 1. Expression of miR-34c-3p and miR-34c-5p in NSCLC tissue(A) miR-34c-3p (left) and miR-34c-5p (right) expression was analyzed in 8 LUAD KRAS wild type patient in TGCA and compared to matched normal tissue. Wilcoxon test was applied to estimate statistical significance ($p = 0.0078$)**.(B)** miR-34c-3p (left) and miR-34c-5p (right) expression was analyzed in 14LUAD $KRAS^{mut}$ patients in TGCA and compared to matched normal tissue. Wilcoxon test was applied to estimate statistical significance ($p < 0.001$)**.**

Supplementary Figure 2. Expression of miR-34c-3p and KRAS protein in NSCLC cell lines and caspase 3 immunofluorescence (A) Expression of miR-34c-3p in several tumor cell lines (A549, HCC827 and Calu-1) and in primary NSCLC cell lines (PT#2, PT#13 and PT#18). MiR-34c-3p expression was assessed by real-time PCR. The transcript levels were normalized over RNU6B expression, used as an internal reference. **(B)** Western blot analysis of KRAS expression upon KRAS cDNA overexpression in HCC827 cell line.**(C)** Immunofluorescence analysis of FITC-conjugated anti cleaved caspase 3 (green) in HCC827 upon miRNA-NC or miRNA-34c overexpression. Nuclei were counterstained with DAPI (blue).

	DNA Change	Type	Consequences	# Affected Cases in Cohort
TCGA-LUAD	chr12:g.25245351C>A	Substitution	Missense KRAS G12C	62 / 202,30.69%
	chr12:g.25245350C>A	Substitution	Missense KRAS G12V	38 / 202,18.81%
	chr12:g.25245350C>T	Substitution	Missense KRAS G12D	20 / 202,9.90%
	chr12:g.25245350C>G	Substitution	Missense KRAS G12A	17 / 202,8.42%
	chr12:g.25245348C>A	Substitution	Missense KRAS G13C	7 / 202,3.47%
	chr12:g.25245351C>T	Substitution	Missense KRAS G12S	5 / 202,2.48%
	chr12:g.25227342T>A	Substitution	Missense KRAS Q61L	3 / 202,1.49%
	chr12:g.25245347C>T	Substitution	Missense KRAS G13D	3 / 202,1.49%
	chr12:g.25245328C>G	Substitution	Missense KRAS L19F	2 / 202,0.99%
	chr12:g.25225628C>G	Substitution	Missense KRAS A146P	1 / 202,0.50%
	chr12:g.25227262T>A	Substitution	Stop Gained KRAS K88*	1 / 202,0.50%
	chr12:g.25245286A>T	Substitution	Missense KRAS D33E	1 / 202,0.50%
	chr12:g.25245328C>A	Substitution	Missense KRAS L19F	1 / 202,0.50%
	chr12:g.25245387T>C	Substitution	5 Prime UTR KRAS	1 / 202,0.50%
	chr12:g.25227341T>A	Substitution	Missense KRAS Q61H	1 / 202,0.50%
TCGA-LUSC	chr12:g.25209820G>C	Substitution	3 Prime UTR KRAS	1 / 44,2.27%
	chr12:g.25225711C>G	Substitution	Missense KRAS C118S	1 / 44,2.27%
	chr12:g.25245350C>A	Substitution	Missense KRAS G12V	1 / 44,2.27%
	chr12:g.25225697T>A	Substitution	Stop Gained KRAS R123*	1 / 44,2.27%
	chr12:g.25245350C>G	Substitution	Missense KRAS G12A	1 / 44,2.27%
	chr12:g.25245348C>A	Substitution	Missense KRAS G13C	1 / 44,2.27%
	chr12:g.25245378C>T	Substitution	Missense KRAS E3K	1 / 44,2.27%
	chr12:g.25245345C>T	Substitution	Missense KRAS V14I	1 / 44,2.27%
	chr12:g.25227341T>A	Substitution	Missense KRAS Q61H	1 / 44,2.27%

Supplementary table 1-KRAS mutational status of 167 TCGA patients. DNA change, type of mutation, and percentage of affected cases is reported.
